# Pubertal Gynecomastia Coincides with Peak Height Velocity

**DOI:** 10.4274/Jcrpe.958

**Published:** 2013-09-18

**Authors:** Yehuda Limony, Michael Friger, Ze’ev Hochberg

**Affiliations:** 1 Ben-Gurion University of the Negev, Faculty of Health Sciences, Pediatric Endocrinology Unit, Beer Sheva, Israel; 2 Meyer Children’s Hospital, Rambam Medical Center, Haifa, Israel; 3 Rappaport Faculty of Medicine and Research Institute, Technion - Israel Institute of Technology, Haifa, Israel

**Keywords:** Insulin-like growth factor-1, gynecomastia, peak height velocity, puberty, growth

## Abstract

**Objective:** Pubertal gynecomastia (PG) occurs in up to 65% of adolescent boys. In this study, we investigated the relationship between the ages at which PG and peak height velocity occur in pubertal boys.

**Methods:** This was a prospective study that was designed to detect PG within three months of its emergence. We examined one hundred and six boys who were followed for short stature and/or delayed puberty at three month intervals, and gynecomastia was observed in 43 of these boys (40.5%).

**Results:** PG occurred in the 43 boys within a year of their peak height velocity, and most of these boys were at Tanner stage 3 for pubic hair and had testicular volumes between 8-10 mL.

**Conclusion:** It is recommended that evaluation of height growth be included in the diagnostic approach to PG in boys with short stature and/or delayed puberty. The coincidence of age of peak height velocity and PG suggests a causal relationship between the two events and a role of insulin-like growth factor-1.

**Conflict of interest:**None declared.

## INTRODUCTION

Benign pubertal gynecomastia (PG) is defined as a benign enlargement of the male breast during puberty, when, according to some studies, the serum estrogen to testosterone ratio is increased (1,2,3). However, it has been reported in two different studies that the sex hormone profile in boys with PG is not different from that of boys without gynecomastia (4,5). In addition, studies in rats have demonstrated that estrogens are ineffective in the absence of insulin-like growth factor-1 (IGF-1) (6).

Benign PG is a distressful and, at times, psychologically disabling condition in adolescent boys, in whom its occurrence can be as high as 65% (7). In view of this high incidence, we believe that an unambiguous definition of benign PG is needed so that it can be distinguished from: (a) PG due to an underlying systemic disorder, such as liver disease, renal failure, or thyrotoxicosis; (b) iatrogenic gynecomastia due to estrogenic drugs, androgen antagonists, anti-ulcer drugs, and chemotherapy; and (c) gynecomastia due to an underlying endocrinopathy, such as Klinefelter syndrome, partial androgen insensitivity, 11-beta hydroxylase deficiency, or 17-ketosteroid reductase deficiency (8).

We have observed that a close time association exists between the emergence of benign PG and the age of peak height velocity (PHV) in pubertal boys. Hence, we postulated that this close time association could be used to distinguish benign PG from the other causes of gynecomastia. This report summarizes our study that was designed to detect benign PG within three months of its emergence, and to correlate its emergence with pubertal stage and age of PHV.

## METHODS

This was a prospective study conducted on 106 pubertal boys who were referred to the Pediatric Endocrinology Unit, Soroka Medical Center, Beer Sheva, Israel because of their short stature, delayed puberty, or both. Since the study was based on data that were acquired during routine clinical practice, informed consent from the research subjects and their parents was not required.

Each boy was clinically examined, and his height was measured every six months when he was at Tanner 1 stage (i.e. before puberty) and every three months during puberty. 

Gynecomastia was defined as uni- or bilateral enlargement of breast tissue greater than 1 centimeter (cm) in diameter. The cut off value of 1 cm was chosen to exclude a clinically doubtful gynecomastia or erected nipple. Gynecomastia that met the inclusion criteria was observed in 43 (40.5%) of the 106 boys referred to the Unit, and these 43 boys comprised the study group. Systemic, iatrogenic, and endocrine diseases were excluded by history, physical examination, and clinical course, and not a single case related to these etiologies was observed in the study cohort. Age at PHV was computed from equations of the Karlberg’s infancy-childhood-puberty growth model (9,10), as previously described (11). The testicular volume of the 106 boys was determined using a Prader orchidometer. Bone age was estimated in only 28 of the 43 boys using the Greulich and Pyle atlas, and was only determined when considered necessary in order to establish the cause of the short stature or delayed puberty. 

## RESULTS

Although some of the boys were referred because of delayed puberty, signs of puberty were observed in all on subsequent follow-up. 

**Figure 1:**
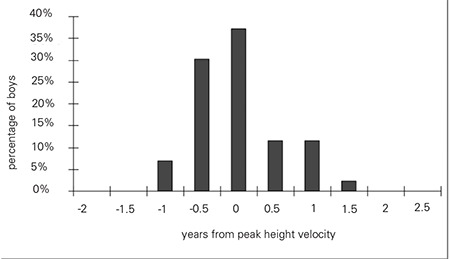
Distribution of the 43 boys according to the time interval between age at peak height velocity (PHV) and age at which gynecomastia was first observed

**Figure 2:**
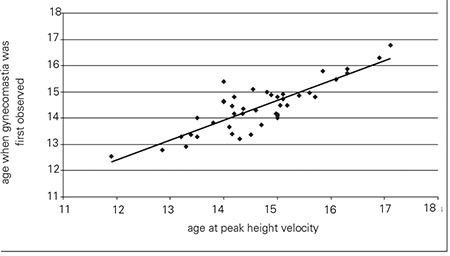
Scatter plot showing a high correlation (r=0.847) between age at which gynecomastia was first observed and age at peak height velocity (PHV)

Gynecomastia was detected when the boys in the study group were 14.4±0.9 years [mean±standard deviation (SD)] old. Their mean age at PHV was 14.7±1.0 years. Forty-two of the 43 boys (98%) developed gynecomastia within a year of their PHV. Figure 1 shows the distribution of the age difference between PHV and gynecomastia. Age of PHV strongly correlated with the age at which gynecomastia was first observed (r=0.847, p<0.0001, Figure 2).

**Figure 3:**
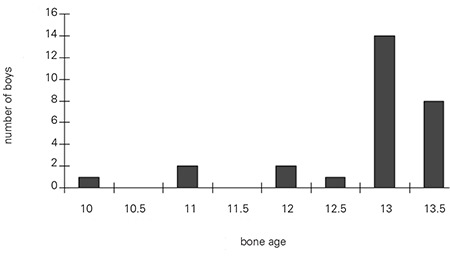
Distribution of the boys according to their bone age when gynecomastia was first observed

**Figure 4:**
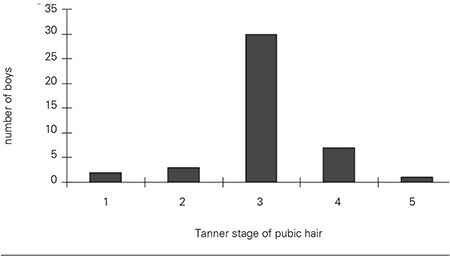
Distribution of the 43 boys according to their stage of pubic hair development when gynecomastia was first observed

When gynecomastia was first observed in the 43 boys, the bone age of 28 boys was 12.8±0.9 years (mean±SD) (Figure 3). Thirty of the 43 boys were at Tanner stage 3 for pubic hair (Figure 4), and testicular volume was between 8.0 and 10 mL when gynecomastia was first observed. 

## DISCUSSION

In an attempt to characterize benign PG in pubertal boys, we have shown in this study that its appearance coincides within a year of age of PHV when the boy is usually at Tanner stage 3 for pubic hair and his testicular volume is between 8-10 mL. 

The selection of the study subjects from a referred group of boys with short stature, delayed puberty, or both may constitute a limitation in the generalization of our results and their inference to boys of normal height and normally timed puberty. On the other hand, we believe that the rigorous physical examinations of the study subjects that were done every three months constitutes the strength of the study.

The concomitant occurrence of gynecomastia and PHV suggests that both events have a common basis. In boys, mid-puberty occurs when they are 13.5-year-old and reach their PHV. This is also the age when serum estradiol levels abruptly increase and serum testosterone levels increase gradually (1). At PHV, serum IGF-1 levels reach their peak, and subsequently decline (12). It has been reported in rats that IGF-1 exerts its maximal effect on the terminal end buds of the mammary gland in the presence of estradiol (6). The persistence of PG beyond mid-puberty occurs mostly in overweight children, who also have elevated serum IGF-1 levels (13). Thus, the concomitant occurrence of PHV and PG suggests that the rise in serum estrogen and IGF-1 levels both influence PHV and the development of PG. The present study is observational; therefore, this hypothesis, in order to be confirmed, needs further investigations that include measurements of blood IGF-1. 

In conclusion, this study shows that benign PG in boys with short stature and/-or with delayed puberty occurs within a year before or after the age of PHV. We therefore recommend that evaluation of height growth be included in the diagnostic approach to a recently discovered PG in these boys. From a practical viewpoint, a follow-up period of three to six months is sufficient to confirm PHV since the height velocity during this period of growth is rapid. Finally, the coincidence of PHV and PG suggests a causal relationship between the two events and a role of IGF-1.
